# Recurrent hepatocellular carcinoma treated by laparoscopic non-anatomical liver resection via the retroperitoneal approach: a case report

**DOI:** 10.3389/fsurg.2026.1826219

**Published:** 2026-05-20

**Authors:** Kecheng Zhang, Yang Liu, Bing Liu, Ruiliang Ge

**Affiliations:** 1 Department of Biliary Tract Surgery, Eastern Hepatobiliary Surgery Hospital, Naval Medical University, Shanghai, China; 2 Department of Urology, Eastern Hepatobiliary Surgery Hospital, Naval Medical University, Shanghai, China

**Keywords:** case report, hepatocellular carcinoma, intraoperative ultrasound, laparoscopic non-anatomical liver resection, retroperitoneal approach

## Abstract

**Background:**

Repeat laparoscopic hepatic resection is an important treatment for recurrent hepatocellular carcinoma. However, intra-abdominal adhesions from prior open surgery and poor exposure of posterior segment lesions significantly increase surgical difficulty and risk. To address these challenges, we adopted an innovative retroperitoneal approach for recurrent segment VI hepatocellular carcinoma after previous open hepatectomy.

**Case presentation:**

We report a case of a patient who had previously undergone open non-anatomical resection of a liver tumor in segment VII, followed by multiple sessions of transarterial chemoembolization and microwave ablation after tumor recurrence. At the 6th year of follow-up after hepatic resection, contrast-enhanced magnetic resonance imaging revealed a recurrent lesion measuring 2.0 × 1.6 cm in segment VI of the liver, adjacent to the adrenal gland. Given the patient's history of prior open surgery and multiple minimally invasive treatments, the possibility of intra-abdominal adhesions was considered. Moreover, the retroperitoneal approach allows direct exposure of the tumor in segment VI of the liver. Therefore, we selected the retroperitoneal approach combined with intraoperative ultrasound and successfully performed a laparoscopic non-anatomical resection of the segment VI liver tumor. The patient recovered smoothly postoperatively and was discharged uneventfully.

**Conclusion:**

For highly selected patients with a small superficial segment VI tumor without major vascular invasion or severe cirrhosis, the retroperitoneal approach for repeat laparoscopic non-anatomical resection is feasible.

## 1 Introduction

Hepatocellular carcinoma (HCC) is the sixth most common malignant tumor globally and the third leading cause of cancer-related mortality worldwide (1). However, the 5-year recurrence rate following radical hepatectomy for HCC remains as high as 50% to 70% (2). Currently, the main therapeutic strategies for recurrent HCC include radiofrequency ablation, transarterial chemoembolization, and liver transplantation, yet reoperation is still recognized as one of the optimal therapeutic approaches (3). In the treatment of recurrent HCC, laparoscopic repeat hepatectomy (LRH) has gradually emerged as a safe and effective minimally invasive option, and several studies have suggested that LRH is feasible in highly selected patients (4–6). Nevertheless, the widespread adoption of LRH continues to face technical challenges, including extensive intra-abdominal adhesions from prior surgery and distorted hilar anatomy, and further evidence is needed to optimize indications and technical standards (7).

For certain cases of recurrent HCC, such as lesions in segments VI and VII, the deep anatomical location and limited operative space caused by intraperitoneal adhesions render the conventional transabdominal laparoscopic approach particularly challenging (8). To address this clinical dilemma, we adopted the technique of retroperitoneal laparoscopic adrenalectomy commonly used in urological practice (9). This retroperitoneal approach effectively avoids potential intra-abdominal adhesions from prior surgery and their negative impact on the surgical field and working space, while allowing more direct exposure of the superficial lesion in segment VI. Herein, we report a case of recurrent HCC in segment VI adjacent to the inferior vena cava and right adrenal gland, successfully resected via the retroperitoneal approach with intraoperative ultrasound guidance.

## 2 Case presentation

### 2.1 Clinical data

**Figure 1:**
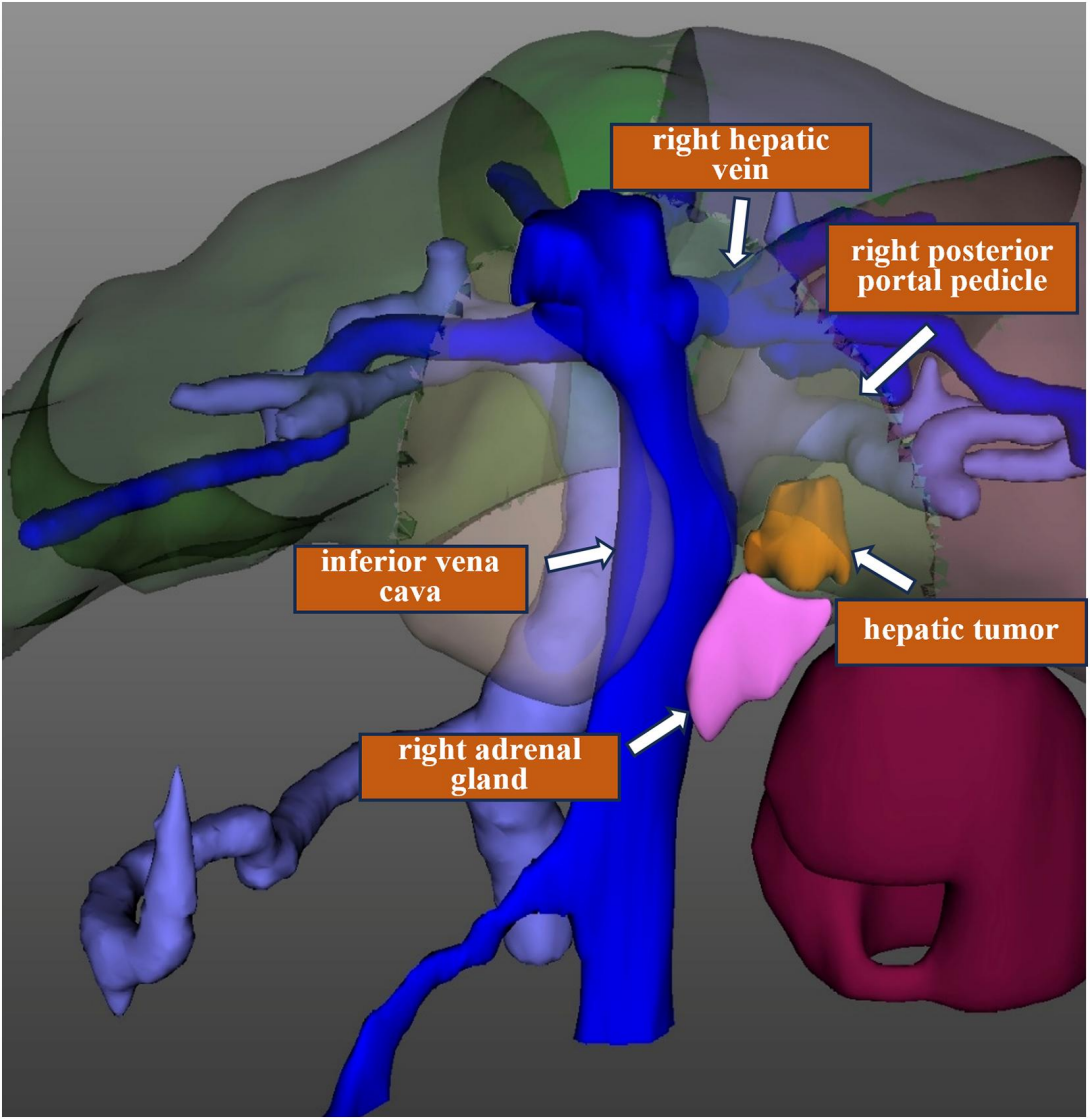
Three-dimensional reconstruction of the recurrent tumor in segment VI. The tumor is close to the inferior vena cava and abuts the right adrenal gland, but is at a distance from the right hepatic vein and the right posterior portal pedicle.

**Figure 2:**
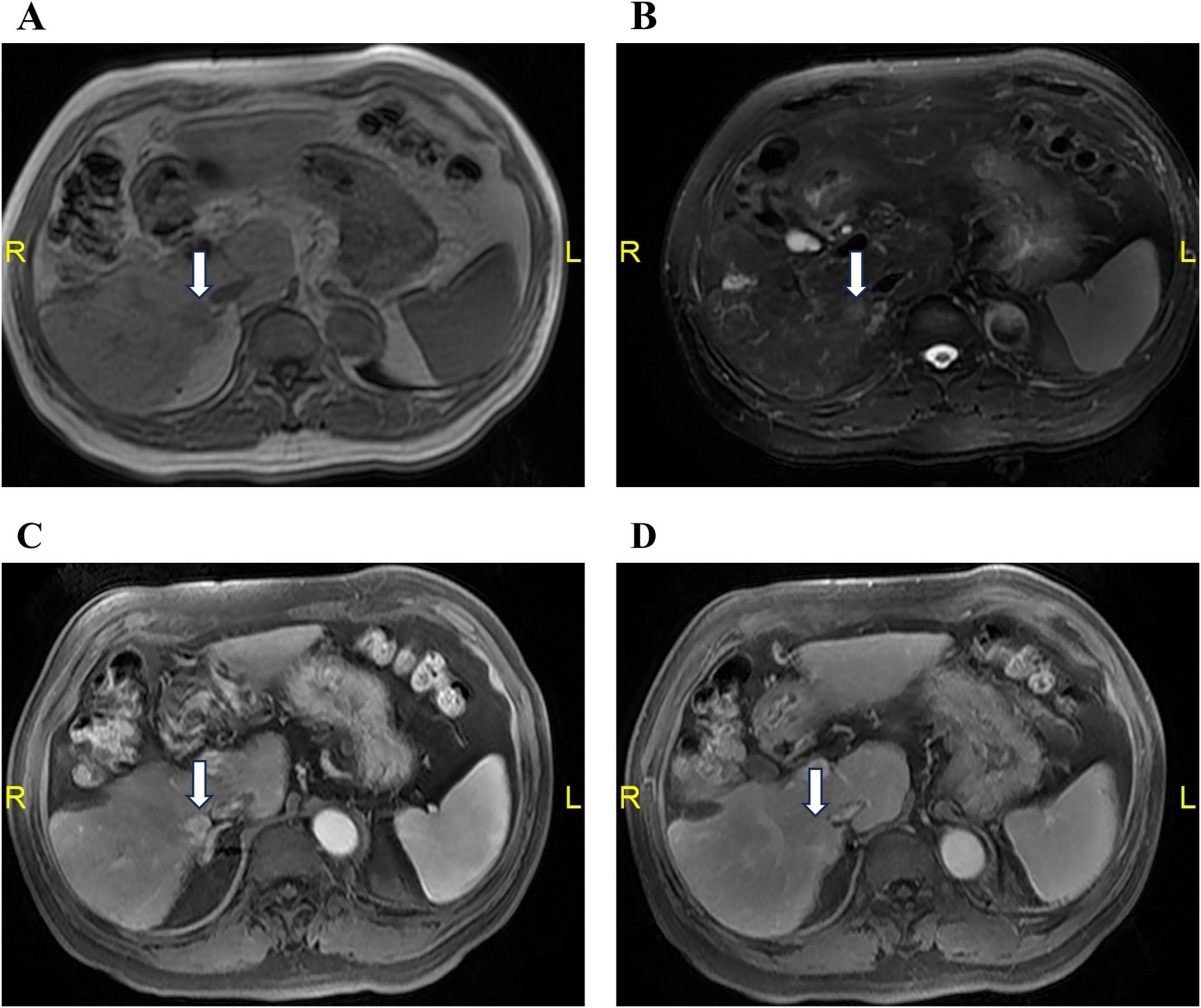
Contrast-enhanced MRI revealed a recurrent lesion in segment 6 of the liver, located superior to the right adrenal gland and to the right of the inferior vena cava. The lesion is hypointense on T1WI **(A)**, hyperintense on T2WI **(B)**, with arterial phase hyperenhancement **(C)** and portal venous phase washout **(D)**.

The patient was a 68-year-old male with chronic hepatitis B virus infection and a positive family history of hepatitis B. He remained asymptomatic, and the recurrent lesion was identified during routine imaging surveillance. He had mild cirrhosis without ascites or esophageal varices (Child-Pugh class A). Laboratory tests showed AFP 6.2 ng/mL (normal <20 ng/mL), PIVKA-II 24 mAU/mL (normal ≤40 mAU/mL), white blood cell count 6.06 × 10⁹/L, platelet count 99 × 10⁹/L, prothrombin time 11.9 s, total bilirubin 22.3 μmol/L, albumin 42 g/L, ALT 36 U/L, and abdominal ultrasound without ascites. According to the Barcelona Clinic Liver Cancer (BCLC) staging system, a solitary 2.0 cm lesion in a patient with Child-Pugh class A liver function and performance status (PS) 0 is classified as BCLC stage 0. He had previously undergone open non-anatomical resection of a liver tumor in segment VII, and postoperative pathology confirmed hepatocellular carcinoma. Due to tumor recurrence, he subsequently underwent 2 sessions of transarterial chemoembolization (TACE) and 3 sessions of microwave ablation (MWA) (Table 1). Six years after the initial HCC resection, a follow-up contrast-enhanced MRI of the liver revealed a new recurrent lesion in the inferior segment of the right liver, measuring 2.0 × 1.6 cm. The tumor was subcapsular in segment VI, closely abutted the adrenal gland, and was located approximately 5 mm from the inferior vena cava (Figure 1). The lesion exhibited hypointensity on T1-weighted images, hyperintensity on T2-weighted images, and a typical “wash-in and wash-out” dynamic enhancement pattern. In accordance with the American College of Radiology (ACR) Liver Imaging Reporting and Data System (LI-RADS), a 2.0 cm lesion with arterial phase hyperenhancement and portal venous phase washout in a high-risk patient with chronic HBV and prior HCC is categorized as LI-RADS 5, indicating definite HCC. This non-invasive diagnosis obviated the need for preoperative biopsy, and the patient proceeded directly to surgical resection (Figure 2). Despite the patient's history of multiple TACE and MWA sessions, surgical treatment was pursued because TACE often fails to achieve complete tumor necrosis, and MWA carried a high risk of thermal injury to the inferior vena cava and adrenal gland due to the proximity of the recurrent lesion. In contrast, repeat hepatectomy offered the possibility of R0 resection with superior local tumor control, and the patient's well–preserved liver function (Child-Pugh A) and solitary recurrence made him a suitable candidate for this approach.

**Table 1:** Timeline of disease course from initial hepatectomy to repeat resection and subsequent follow-up.

Time point	Clinical event	Details
May 2016	Initial open non-anatomical resection of right liver tumor	Initial diagnosis of HCC in segment VII, tumor size 2.5 × 2.0 cm
June 2016	Prophylactic transarterial chemoembolization	Uneventful postoperative recovery
March 2020	Transarterial chemoembolization	Recurrent lesion in segment III treated with TACE
April 2020	Microwave ablation	Residual viable lesion in segment III treated with MWA
July 2020	Microwave ablation	Recurrent lesion in segment VIII (1.9 × 1.7 cm) treated with MWA
February 2021	Microwave ablation	New recurrent lesion in segment VI treated with MWA
June 2022	Repeat laparoscopic non-anatomical resection of right liver tumor via retroperitoneal approach	New recurrent HCC in segment VI (2.0 × 1.6 cm); R0 resection achieved; uneventful recovery
May 2024	Follow-up (23 months post-repeat resection)	New recurrence in segment IV; treated with MWA

### 2.2 Surgical information

**Figure 3:**
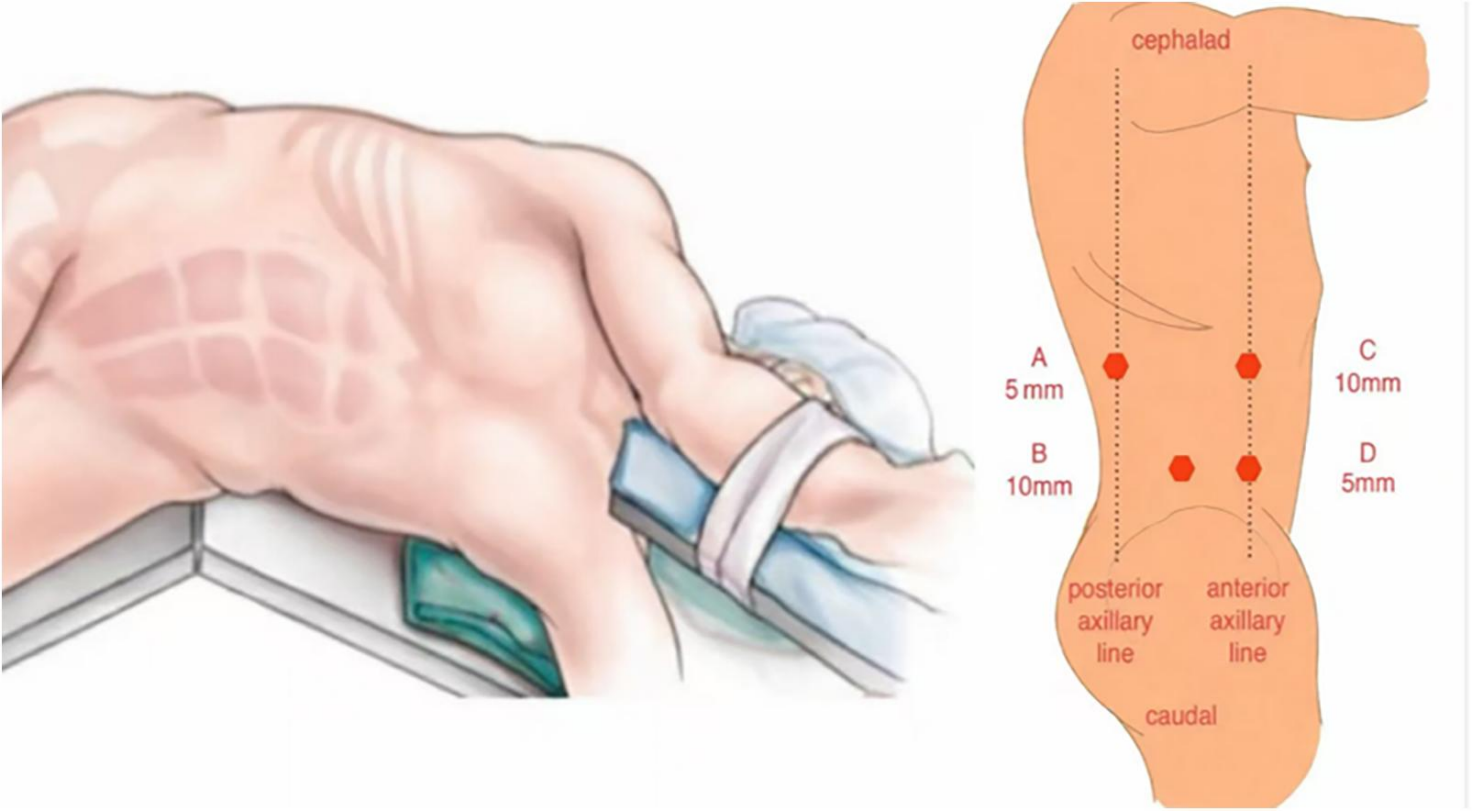
Schematic illustration of trocar placement for the retroperitoneal approach. Port B (10 mm) is located at the intersection of the right iliac crest and the right midaxillary line. Port C (10 mm) is placed at the right anterior axillary line. Port A (5 mm) is placed at the right posterior axillary line. Port D (5 mm) is placed 5 cm below the costal margin at the right anterior axillary line.

**Figure 4:**
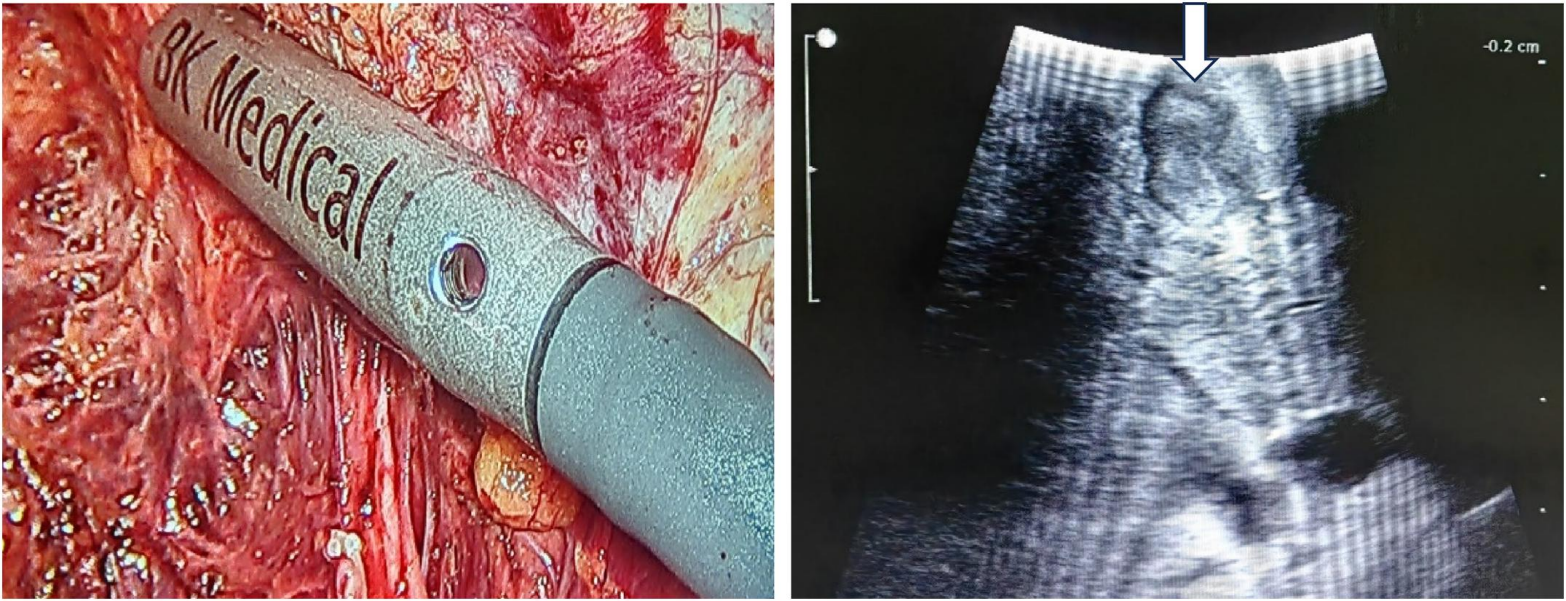
Intraoperative ultrasound delineating the tumor margin and guiding the resection line.

The patient was placed in a full left lateral decubitus position with a 90° lateral tilt. The operating table was flexed at the iliac crest level to widen the costal–iliac distance and thus enlarge the retroperitoneal working space. The patient was secured with a gel pad and further fixed to the table using wide adhesive tape across the hips and shoulders. The right flank was elevated, and the patient was positioned with the iliac crest aligned to the break of the operating table. An incision was made at the intersection of the right iliac crest and the right midaxillary line. The fat space around the incision was expanded to place a Trocar. Carbon dioxide gas was insufflated to a pressure of 13 mmHg. A laparoscopic lens was then inserted. Under direct vision, a 1–cm incision was made under the costal margin of the right anterior axillary line. A 0.5-cm incision was made under the costal margin of the right posterior axillary line. Another 0.5-cm incision was made 5 cm below the costal margin of the right anterior axillary line. Trocars and operating instruments were inserted through these incisions (Figure 3). The retroperitoneal working space was developed using a technique adapted from retroperitoneoscopic adrenalectomy. After skin incision, blunt finger dissection and balloon dilation were used to create the space. The plane between the liver and the adrenal gland was identified by blunt dissection along the natural avascular areolar tissue, with the kidney, adrenal gland, and inferior vena cava serving as key landmarks. The peritoneum was avoided by staying posterior to it and using low CO₂ insufflation pressure. During the operation, the perirenal fat on the right side was carefully dissected. This dissection exposed the right adrenal gland, the adjacent inferior vena cava, and the tumor on the capsular surface of segment VI of the liver. Intraoperative ultrasound (IOUS) was used at the outset to identify the tumor, delineate the resection margin, and determine the extent of resection. During parenchymal transection, IOUS was applied intermittently to monitor the relationship between the transection plane and the adjacent vessels and bile ducts, and to ensure an adequate safety margin (Figure 4). The liver was incised using an ultrasonic scalpel, and important ducts were ligated with Hem-o-lok clips. After complete transection, absorbable hemostatic powder was sprayed onto the cut surface. The specimen was placed in an endoscopic retrieval bag and extracted intact. The resected liver specimen measured approximately 4.0 × 4.0 × 3.2 cm; the formalin-fixed tumor measured 2.0 × 1.6 × 1.2 cm. Controlled low central venous pressure was applied as the primary strategy to reduce intraoperative bleeding, given that the Pringle maneuver was not feasible. The target CVP was maintained between 3 and 5 cmH₂O, with mean arterial pressure kept ≥60 mmHg. Fluid administration was strictly restricted during the pre-transection phase until completion of liver parenchymal transection; after transection, crystalloids and colloids were rapidly replenished. The anesthesiologist continuously monitored CVP and MAP and adjusted the infusion rate of vasoactive agents accordingly. The surgery was completed successfully, with an operative time of 130 min and an estimated intraoperative blood loss of approximately 50 ml.

### 2.3 Pathology results

Microscopic findings: The cancerous tissues were arranged in a trabecular pattern. The tumor cells were polygonal in shape with large, hyperchromatic nuclei and marked atypia. Focal lymphocytic infiltration was observed within the tumor. A partial fibrous capsule was present at the tumor periphery. Vascular invasion was identified. Resection margins were negative for tumor cells, with a distance of 0.4 cm from the tumor to the margin. The background liver showed no pseudolobule formation, with mild macrovesicular steatosis of hepatocytes.

Pathological diagnosis: Hepatocellular carcinoma, trabecular type, grade Ⅲ, microvascular invasion (MVI)=M1.

Immunohistochemistry: Hep-1(+), Arginase(+), GPC3(+), CK19(-), BSEP(+), CD34(+), Ki67(25%+).

### 2.4 Postoperative follow-up

The patient resumed oral intake and ambulation on postoperative day (POD) 1 and was discharged uneventfully on POD 5. At 23 months postoperatively (May 2024), contrast-enhanced MRI revealed a new recurrent lesion in segment IV. The patient subsequently underwent microwave ablation of this lesion.

## 3 Discussion

Laparoscopic hepatectomy is one of the most important advances in minimally invasive techniques in the field of hepatobiliary surgery over the past three decades. Initially, this technique was only applicable to simple resection of superficial benign hepatic lesions. It has now gradually developed into a mature and widely recognized minimally invasive surgical method for the treatment of benign and malignant hepatic diseases. Nevertheless, for patients with recurrent hepatocellular carcinoma who have a history of open surgery and severe intra-abdominal adhesions, the conventional transabdominal route often becomes difficult because of distorted anatomy and limited working space. This case offers an alternative strategy: a retroperitoneal approach for laparoscopic non-anatomical resection of a recurrent segment VI HCC, combined with controlled low central venous pressure and intraoperative ultrasound. The main advantage of this approach is that it effectively avoids peritoneal adhesions and gives direct access to a superficial segment VI lesion lying close to the adrenal gland and the inferior vena cava. It must be acknowledged that for most hepatobiliary surgeons, the transabdominal route remains the standard choice for resecting a segment VI lesion, and its safety and feasibility are well documented. In this particular case, our decision to use the retroperitoneal approach was made preoperatively on empirical grounds-to bypass possible adhesions and to directly expose the target lesion. We did not attempt a transabdominal approach. Hence, we emphasize that the retroperitoneal route should be seen only as an alternative for highly selected patients. For the majority of patients, the transabdominal route remains the standard.

According to the operative space, the surgical approaches of laparoscopic hepatectomy can be classified into the transabdominal approach, retroperitoneal approach, transthoracic approach, or thoracoabdominal approach, among others. The majority of laparoscopic hepatectomies are performed via the transabdominal approach, which provides a broad surgical field and conforms to the operating habits of conventional open surgery. The transthoracic approach is suitable for selected subphrenic hepatic lesions, particularly in patients with severe intra-abdominal adhesions, and offers a more direct route but requires one-lung ventilation. The thoracoabdominal approach combines transabdominal hilar occlusion with transthoracic exposure and may also be considered in similar settings (10, 11). The retroperitoneal approach provides direct access to the bare area of the liver and is generally indicated for the resection of lesions in segments 6 and 7 of the right posterior liver; due to the deep location, poor exposure, and unfavorable operating angles of tumors in this region, it is particularly suitable for patients with a history of abdominal surgery and severe intra-abdominal adhesions (12). As the retroperitoneal approach does not allow for Pringle maneuver, it is suitable for the resection of small, superficially located liver tumors, and urological surgeons are often required to assist in establishing the access route (13). In this setting, controlled low central venous pressure (CLCVP) is especially valuable for minimizing blood loss and maintaining a clear operative field (14–16).

The technique draws on urological experience with the retroperitoneal laparoscopic approach, but it requires specific training for hepatobiliary surgeons. The learning curve is steep, as the operator must become familiar with the reverse spatial orientation typical of retroperitoneal anatomy. We therefore recommend that a surgical team accumulate prior experience in conventional laparoscopic hepatectomy and retroperitoneal adrenalectomy before attempting this approach. Based on the relevant literature on retroperitoneal laparoscopic hepatectomy, we summarize that the following considerations should be emphasized when selecting patients for this approach: tumor diameter <3 cm; location in the postero-inferior part of segment VI or VII, superficial (depth ≤2 cm) or protruding from the liver capsule; no major vascular invasion; Child-Pugh class A liver function without clinically significant portal hypertension; and operator experience with both conventional laparoscopic hepatectomy and retroperitoneal approaches (12). This technique might also be considered for other tumor types, such as colorectal liver metastases in posterior segments, although further cases are needed for validation (17).

Several limitations should be noted. First, this is a single case, and no oncological conclusions can be drawn from it. Of particular note, the recurrence in segment IV at 23 months postoperatively occurred in the setting of grade III HCC with microvascular invasion (MVI, M1) and a narrow 4 mm resection margin, which is consistent with the known aggressive biology of such tumors. It remains unclear whether this recurrence represents clonal spread from the segment VI tumor or a *de novo* second primary in the HBV-damaged liver, as this distinction cannot be determined from the available data. Furthermore, while the resection margin was technically negative, a 4 mm margin may be oncologically inadequate for an MVI-positive tumor. Accumulating evidence suggests that for patients with MVI-positive HCC, anatomical hepatectomy with a wide resection margin (≥1 cm) may positively influence long-term prognosis (18). It should be acknowledged, however, that the proximity of the lesion to the inferior vena cava imposed an inherent anatomical constraint, and a wider margin would likely have been unattainable regardless of the surgical approach employed. Nevertheless, whenever anatomically feasible, an operative strategy that permits a more generous resection margin should be pursued, as this may confer particular benefit in patients with MVI-positive, grade III recurrences. Second, the absence of an intraoperative video limits the visual communication of the technique. Third, the decision to use the retroperitoneal route was based on clinical judgment rather than objective evidence of adhesions. Fourth, the existing literature on retroperitoneal laparoscopic hepatectomy consists of only a few case reports, with no systematic reviews or comparative studies available.

As laparoscopic techniques and instruments continue to evolve, the combination of intraoperative ultrasound and CLCVP may help broaden the applicability of the retroperitoneal approach. Future studies might also explore whether a hybrid strategy combining transabdominal hilar occlusion with retroperitoneal resection could overcome the current inability to perform the Pringle maneuver, although this concept remains purely speculative at present. Nevertheless, the long-term oncological outcomes of the retroperitoneal approach need to be assessed in larger patient series with extended follow-up.

## 4 Conclusions

This case demonstrates that, in highly selected patients with a small superficial tumor in segment VI, no major vascular invasion, and no severe cirrhosis, the retroperitoneal approach for repeat laparoscopic non-anatomical resection is a feasible surgical option. The combination of CLCVP and IOUS can partially compensate for the inability to perform the Pringle maneuver, but this approach remains limited to carefully selected patients, and its safety and long-term outcomes warrant further study.

## 5 Patient perspective

The patient was informed of his diagnosis and the available treatment options, including TACE, MWA, and surgical resection. He was concerned that repeated TACE or MWA might result in incomplete tumor necrosis and suboptimal local efficacy. After a detailed discussion of the risks and benefits of each option, he strongly preferred surgical resection to achieve a potential cure. Following the procedure, he reported a smooth postoperative recovery with minimal pain and a short hospital stay. He was able to resume normal daily activities after discharge and experienced no significant impact on his quality of life.

## Data Availability

The original contributions presented in the study are included in the article/Supplementary Material, further inquiries can be directed to the corresponding author/s.
